# Tuning the Swelling Behavior of Superabsorbent Hydrogels with a Branched Poly(aspartic acid) Crosslinker

**DOI:** 10.3390/gels11030161

**Published:** 2025-02-24

**Authors:** Sunggyu Shin, Sangjin Kim, Sukhyeon Hong, Namhyun Kim, Juhwan Kang, Jaehyun Jeong

**Affiliations:** Department of Chemical Engineering, Soongsil University, Seoul 06978, Republic of Korea; whitegd45@soongsil.ac.kr (S.S.); sj1229v@gmail.com (S.K.); hsh4768@soongsil.ac.kr (S.H.); asxz7505@soongsil.ac.kr (N.K.); goraeme3@naver.com (J.K.)

**Keywords:** branched crosslinker, superabsorbent hydrogel, poly(aspartic acid), swelling kinetics

## Abstract

Superabsorbent hydrogels used in products like diapers, hygiene items, and medical patches depend on their swelling ratio. However, improving the swelling performance across hydrogel assemblies remains challenging. This study identifies a decline in the water absorption capacity in hydrogel assemblies with high swelling ratios, as confirmed through MRI analysis, and introduces a solution using a branched crosslinker to address this issue. The branched crosslinker was synthesized by grafting acrylate groups onto poly(aspartic acid)s. This branched poly(aspartic acid) crosslinker was incorporated into hydrogels with the same number of acrylate groups as PEGDA575, a conventional linear crosslinker, and their absorption performance and behavior were compared. The results showed that hydrogels with the branched crosslinker exhibited a swelling ratio twice as high as the PEGDA575 group, with a slower initial absorption rate, demonstrating a more gradual swelling behavior. Additionally, while the initial absorption rate was approximately 30% slower than the PEGDA575 group, the absorption rate showed a gradual decrease of less than 15% within the first 30 min, indicating sustained absorption behavior. Overall, the new strategy presented in this study of introducing a branched crosslinker into hydrogels is expected to be a useful application for existing industries by enhancing swelling ratios and promoting continuous absorption.

## 1. Introduction

Superabsorbent hydrogels crosslinked with hydrophilic polymers can absorb solvents up to tens or hundreds of times their own weight. Due to this remarkable ability, they have been widely applied in various fields, including drug delivery systems, biomaterials engineering, and medicine [1,2,3,4,5]. Traditionally, such hydrophilic hydrogels have been used in industries producing goods such as soil conditioners, diapers, and sanitary products [6,7,8,9,10]. Hydrogels can be easily tailored for specific mechanical strengths and swelling behaviors by adjusting factors like the type and concentration of hydrophilic polymers and the degree of crosslinking during the pre-gel solution phase [11,12,13]. Recently, the analysis of the absorption performance of hydrogel assemblies has also been conducted, employing methods like the “tea bag method” [14,15,16]. Furthermore, research on the swelling behavior of hydrogels under various environmental conditions has been actively conducted, particularly using highly hydrophilic polymers like poly(acrylic acid) and creating micro-sized particles to either absorb water instantly or retain water for extended periods [17,18,19].

However, while the traditional methods for evaluating hydrogel swelling performance—such as measuring the swelling ratio—provide valuable insights into swelling behavior [20,21], they have limitations in fully representing the overall absorption behavior of hydrogel assemblies. Similarly, although the “tea bag method” can demonstrate the swelling performance of hydrogel assemblies based on their mass, it does not adequately describe the real-time swelling behavior of the assemblies. These limitations highlight the need for further research to better understand the absorption behavior of hydrogel assemblies. Therefore, MRI has been noted as a promising technology for real-time monitoring of hydrogel swelling behavior, given the limitations of the traditional methods for evaluating hydrogel swelling performance. A new approach to controlling the properties of hydrogels involves the synthesis of branched crosslinkers for use in hydrogel networks [22,23]. The synthesized crosslinkers allow for control over the number of acrylate groups per unit length in the polymer backbone. These acrylates participate in the hydrogel polymerization process, linking with monomer chains to provide crosslinking. Recent studies have shown that branched crosslinkers can decouple the dependent relationship between mechanical properties and swelling performance, enabling independent control over each aspect [22,24].

**Figure 1:**
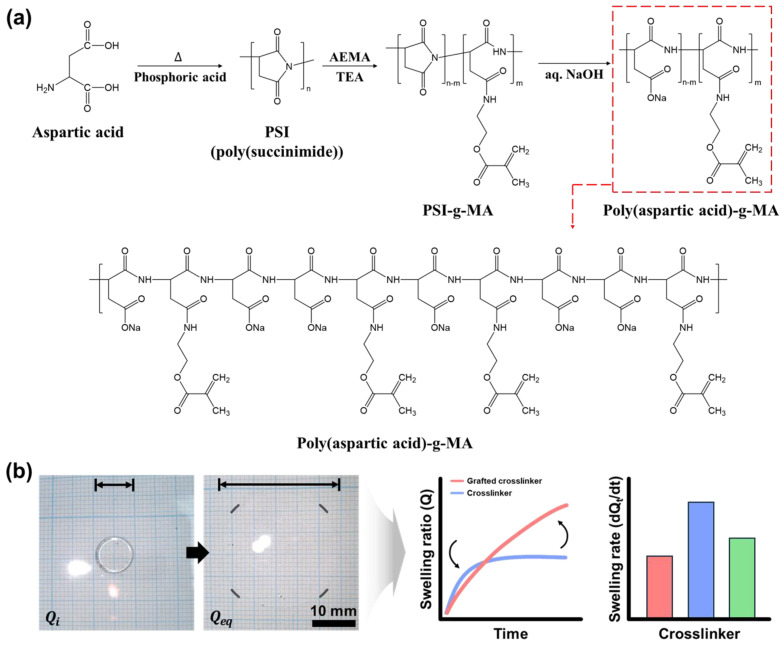
Swelling properties of hydrogels with the introduction of branched crosslinkers. (**a**) Schematic representation of the synthesis of methacrylate conjugated poly(aspartic acid)(PAsp-g-MA) branched crosslinker. (**b**) The branched crosslinker is used as a crosslinking agent in hydrogels to tune their swelling capacity and swelling behavior.

This study proposes a branched poly(aspartic acid) crosslinker as an alternative to conventional linear crosslinkers for superabsorbent hydrogels. While previous studies have explored various crosslinking strategies, the application of a branched poly(amino acid)-based crosslinker has not been reported in the context of superabsorbent hydrogels (Figure 1). This crosslinker design offers a new method to control hydrogel swelling behavior independently from mechanical properties, which has been a long-standing challenge in the field. Additionally, by employing MRI-based real-time absorption analysis, this study provides novel insights into the swelling behavior of hydrogel assemblies rather than just individual particles. The hydrogel assemblies with a high swelling ratio do not necessarily achieve optimal absorption in practical applications, and this phenomenon that has not been well documented. MRI analysis is used to identify the issues in the behavior of conventional hydrogel aggregates, further demonstrating that by synthesizing a branched crosslinker, such as branched poly(aspartic acid), the water absorption behavior of the hydrogels can be modified and improved. This new approach of using a branched crosslinker directly addresses this issue by enhancing overall absorption efficiency in hydrogel assemblies, making it highly relevant for industrial applications such as medical patches, hygiene products, and biomaterials.

## 2. Results and Discussion

### 2.1. Absorption Behavior of Hydrogel Particle Assemblies

**Figure 2:**
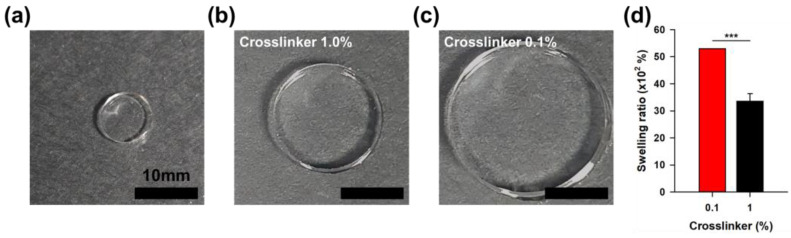
Swelling behavior of hydrogels as a function of crosslinker content. (**a**) Hydrogels were punched into 8 mm discs immediately after preparation and then immersed in 0.9% saline for 24 h to achieve full swelling. (**b**) PEGDA575 1.0%, and (**c**) PEGDA575 0.1% (Scale bar: 10 mm). (**d**) Graph showing the difference in swelling ratios as a function of PEGDA575 content.

Acrylic acid-based hydrogels are widely utilized in various applications such as consumer products, industrial goods, and medical devices due to their excellent absorption properties. To enhance absorption capacity, methods such as lowering the crosslinking density or increasing the concentration of acrylic acid are commonly employed. In this study, we prepared hydrogels by crosslinking 70 mol% neutralized acrylic acid (32.5% *w*/*w*) with 0.1% and 1.0% (*w*/*w*) PEGDA575 relative to acrylic acid content and analyzed their swelling ratios when immersed in saline (NaCl aq. 0.9%) (Figure 2). The swelling ratio at full expansion for the hydrogel with 0.1% crosslinker was 5300%, which was 58% higher compared to 3360% for the hydrogel with 1.0% crosslinker (Figure 2d). This behavior aligns with that observed in typical hydrogels and demonstrates that using hydrogels with a high swelling ratio is a strategically advantageous choice in various industries.

**Figure 3:**
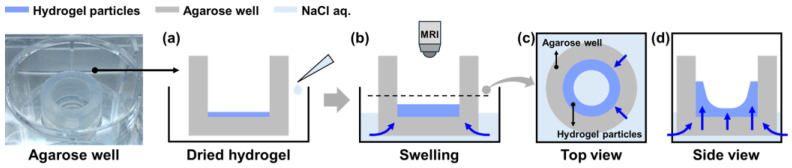
MRI Schematic for Evaluating the Absorption Behavior of Hydrogel Particle Assemblies in an Agarose Well. (**a**) Hydrogel particle assemblies are placed into the agarose well. (**b**) The agarose well is immersed in 0.9% saline, and swelling is monitored using MRI. The saline passes through the agarose well and reaches the hydrogel particle assemblies. MRI images show the (**c**) top view and (**d**) side view of the particle assemblies, enabling the analysis of water movement.

Hydrogels crosslinked with PEGDA575, a conventional linear crosslinker, were polymerized and dried, and the real-time water absorption behavior of hydrogel particle assemblies was observed using MRI. To provide a controlled environment for water exposure, rather than direct contact with water, an agarose well was introduced. The results revealed imbalanced absorption behavior in hydrogel assemblies with high swelling ratios [24,25]. The dried hydrogel particles absorbed water on the agarose well surface, and the absorption behavior was observed by examining the vertical and horizontal cross-sections every 10 min (Figure 3).

**Figure 4:**
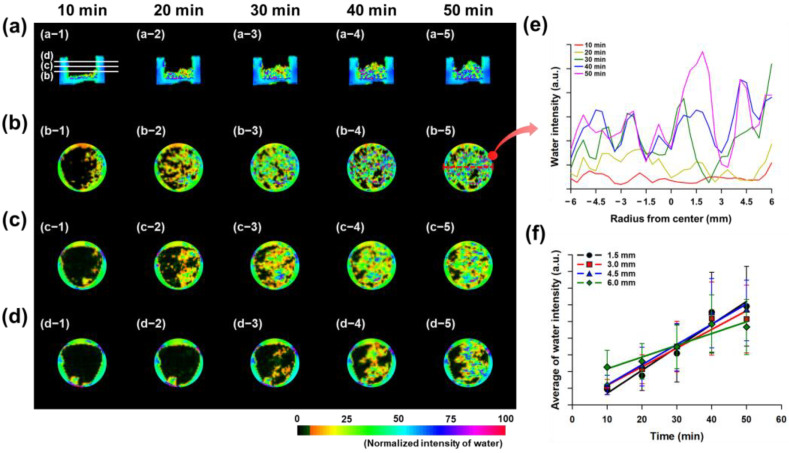
MRI assessment of swelling in hydrogel particle assemblies crosslinked with 1.0% PEGDA575. (**a**) The side view shows the movement of water toward the upper layer as the hydrogel particle assemblies swell. (**b**) The hydrogel swells from the bottom upwards, and (**c**,**d**) further expands towards the upper layer over time. (**e**) Hydrogel particles in the same layer absorbed a relatively uniform amount of water over time. (**f**) A consistent amount of water absorption was observed across different distances from the center of the agarose well.

Figure 4a–e present representative images and illustrate the water absorption behavior of hydrogel particles at different positions. The data obtained from these images were measured multiple times, and the average water absorption results based on position are given in Figure 4f. The results showed that the hydrogel with 1.0% PEGDA575 absorbed water and gradually swelled, exhibiting an overall upward movement of water (Figure 4a). As time progressed, the hydrogel particles absorbed water and swelled, pushing the swollen hydrogels upwards and transporting water to the upper layers (Figure 4b–d). Within the same layer, the hydrogel particles absorbed similar amounts of water at the same time, and the amount of absorbed water increased as time elapsed (Figure 4e). As shown in Figure 4e, which represents the results for hydrogels with a crosslinking density of 1.0%, water is initially absorbed more rapidly in the peripheral regions of the disc (where water first enters) before gradually penetrating toward the center, leading to an overall uniform distribution across the cross-section. Furthermore, even within a certain distance from the center, both the amount of water absorbed and the rate of absorption increased similarly over time (Figure 4f).

**Figure 5:**
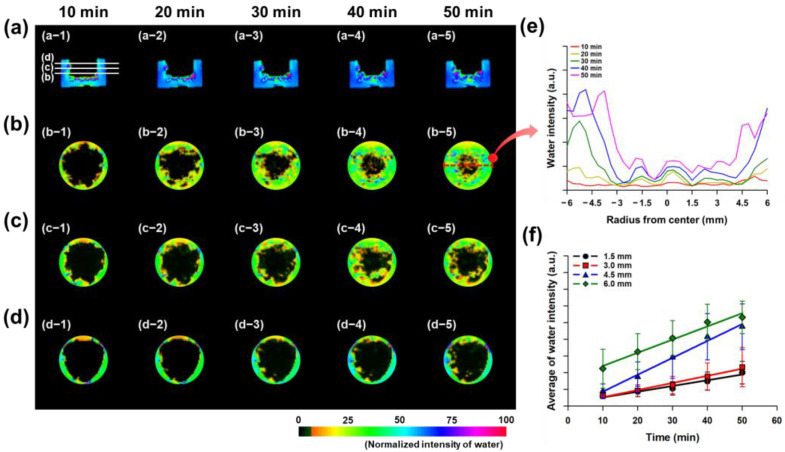
MRI assessment of swelling in hydrogel particle assemblies crosslinked with 0.1% PEGDA575. (**a**) The swelling of the hydrogel particle assemblies resulted in water moving toward the upper layer; however, the expansion was more pronounced closer to the agarose well walls than at the center. (**b**) Greater water signal was observed near the agarose well walls, and (**c**,**d**) over time, the hydrogel closer to the walls expanded further towards the upper layer. (**e**) In the same layer, hydrogel particles near the center showed less swelling, whereas those closer to the agarose well walls expanded more. (**f**) The area near the center of the agarose well exhibited slower water absorption compared to the areas farther from the center.

In contrast, although the hydrogel with 0.1% PEGDA575 exhibited a high swelling ratio, the absorption of water was slower compared to the 1.0% PEGDA575 hydrogel, and uneven absorption behavior was observed, with greater swelling at the edges than in the center (Figure 5a). The hydrogel particles that absorbed water exhibited a similar upward displacement as in the 1.0% PEGDA575 hydrogel; however, they swelled more quickly near the agarose well surface (Figure 5b–d). Over time, the water absorption of the hydrogel particles tended to increase, but the amount of absorbed water was lower closer to the center (Figure 5e). Additionally, the water absorption gradually increased closer to the center, while absorption was faster as the distance from the center increased (Figure 5f). The MRI results presented in Figure 4 and Figure 5 specifically focus on the extreme cases (0.1% and 1.0%) to highlight the potential issues in hydrogel particle assemblies under the conditions of high and low swelling. This selection was made to clearly demonstrate the challenges associated with swelling imbalance in hydrogel assemblies and to emphasize the advantages of the branched crosslinker system.

The purpose of the MRI analysis in this study was to investigate the actual water absorption behavior and the movement of water within hydrogel particle aggregates, which are commonly used in hydrogel systems. The aim was not only to examine the swelling ratio but also to highlight the importance of considering other characteristics, such as the initial swelling rate and the maintenance of swelling rate. This was carried out to emphasize that these aspects cannot be fully addressed by simple changes in the concentration of conventional linear crosslinkers. These results suggest that the hydrogel particles at the boundaries absorbed water first, and water transfer within the particle assembly was hindered. In other words, even though the hydrogel with lower crosslinking density and high swelling ratio absorbs water and swells initially, it may block the spaces between the hydrogel particles or prevent water from being transferred to the adjacent hydrogel particles, impeding the movement of water within the particle assembly. Traditionally, the introduction of hydrogels with a high swelling ratio has been proposed, as they demonstrate superior absorption properties and can enhance the swelling performance of a system [26]. However, for hydrogel particle assemblies, it is essential to regulate the initial absorption rate, as excessive absorption in individual hydrogels can reduce the overall absorption capacity of the assembly. Therefore, MRI analysis was used to identify issues in the behavior of conventional hydrogel aggregates, and we demonstrated that by synthesizing a branched crosslinker, such as branched poly(aspartic acid), we can modify and improve the water absorption behavior of a hydrogel. In short, the MRI analysis was employed to visualize these problems, and the results demonstrated that the synthesized crosslinkers could effectively address these issues by modifying the swelling behavior and other relevant characteristics.

### 2.2. Structural Characterization of Branched Crosslinker, PAsp-g-MA

The hydrophobic PSI (Mw: 19,000 g/mol) was synthesized by grafting methacrylate based on the degree of substitution, as summarized in Table 1. The samples were named PSI-M20 and PSI-M30, where the degree of grafting referred to the number of hydrophobic substituents per 100 repeating units of PSI. The synthesized PSI-g-MA was characterized using ^1^H-NMR, where the methylene protons of the succinimide unit were observed at 2.7 ppm and 3.2 ppm, the methine proton at 5.3 ppm, the methylene protons of the introduced acrylate appeared at 5.6 ppm and 6.0 ppm, and the methyl protons at 1.87 ppm. The degree of substitution (DS) was calculated using the integral values of the characteristic peaks of PSI (-CH) and MA (-CH_3_) and was calculated using the following formula. The results of the actual DS calculation confirmed that 13% and 17% of substitution were achieved. The synthesized PSI-g-MA was mixed with NaOH solution and further polymerized into poly(aspartic acid)-g-MA, which was ultimately used as the branching crosslinker in this study.

(1) $${DS}_{MA}\;\left(\%\right)=\frac{The\;integral\;of\;the\;peak\;1.8\sim 1.9\;ppm/3}{The\;integral\;of\;the\;peak\;5.1\sim 5.5\;ppm/3}\times 100$$

### 2.3. Swelling Behavior of Hydrogels with Branched Poly(aspartic acid) Crosslinkers

**Figure 6:**
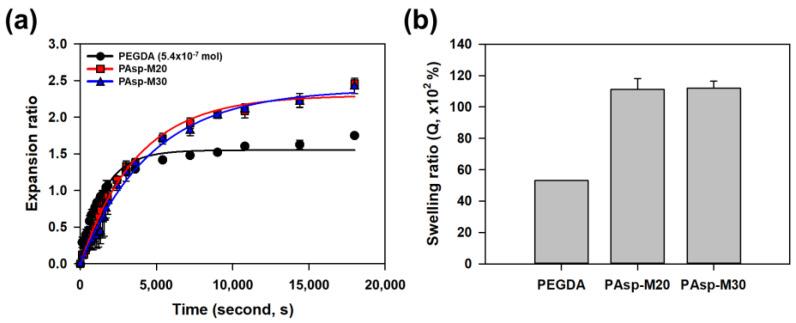
Real-time swelling behavior of hydrogels. (**a**) Hydrogels crosslinked with branched crosslinkers exhibited a higher swelling ratio compared to those crosslinked with PEGDA575 0.1%. (**b**) Hydrogels with branched crosslinkers were able to absorb a larger volume of water per unit weight compared to those crosslinked with PEGDA575.

Acrylic acid, neutralized to 70 mol%, was polymerized with both PEGDA575 and the synthesized branched poly(aspartic acid) crosslinker, PAsp-g-MA, to form hydrogels. To systematically investigate the effect of crosslinking density, the crosslinker content was varied across four levels (0.1, 0.4, 0.7, and 1.0%) and the swelling behavior of the hydrogels was examined (Table 2). The hydrogels were then immersed in a saline solution, and the real-time swelling behavior was monitored over a period of 5 h. As a result, the hydrogels with PAsp-M20 and PAsp-M30 exhibited 1.5 times greater swelling than those crosslinked with PEGDA575, even though the same amount of acrylate (5.4 × 10^−7^ mol) was used (Figure 6a). Additionally, the swelling ratios of the PAsp-M20 and PAsp-M30 hydrogels were 110 × 10^2^% and 112.0 × 10^2^%, respectively, significantly higher than the 53 × 10^2^% swelling ratio of the PEGDA575 0.1% hydrogels (Figure 6b). The results indicated that hydrogels with the synthesized branched poly(aspartic acid) crosslinker, PAsp-g-MA, demonstrated superior water absorption compared to the PEGDA575-crosslinked hydrogels, even with the same amount of acrylates.

**Figure 7:**
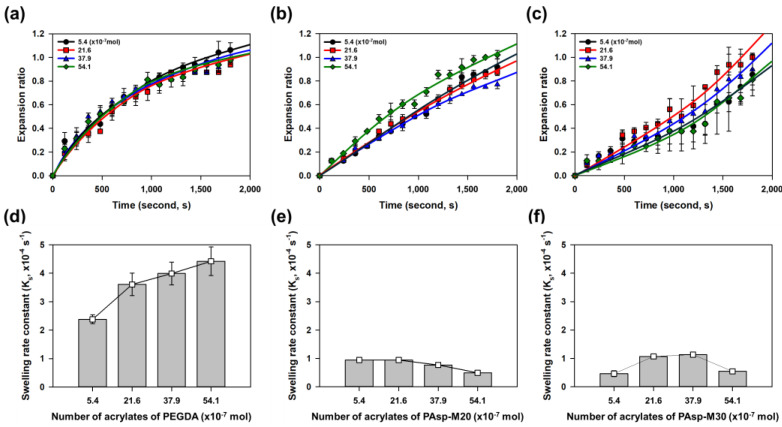
Analysis of initial absorption behavior of hydrogels. (**a**) Hydrogels crosslinked with PEGDA575 exhibited a tendency for rapid initial swelling. As the degree of substitution (DS) of the branched crosslinker increased, the initial swelling of hydrogels crosslinked with branched crosslinkers became slower compared to those crosslinked with PEGDA575 (**b**,**c**). (**d**) The swelling rate constant of PEGDA575-crosslinked hydrogels tended to increase as the crosslinker content increased. (**e**,**f**) Hydrogels incorporating branched crosslinkers showed much lower swelling rate constant values than those crosslinked with PEGDA575, with little variation according to the crosslinker content.

To assess the initial absorption behavior, the swelling ratios due to water absorption were monitored during a time window between 0 and 1800 s (Figure 7a–c). As a result, the hydrogels with branching crosslinkers exhibited more gradual swelling compared to the PEGDA575-crosslinked hydrogels, which displayed a faster initial increase in swelling (Figure 7a). The increase in swelling was slower in the PAsp-M30 hydrogels compared to the PAsp-M20 hydrogels, which is consistent with the higher degree of substitution in PAsp-M30 (Figure 7b,c). Consequently, by incorporating the branching crosslinker, the swelling ratio of the hydrogels increased while allowing for a more controlled initial expansion. This behavior was analyzed using the swelling rate constant. The swelling rate constant for the PEGDA575-crosslinked hydrogels, which exhibited relatively low swelling ratios, ranged from 2.4 × 10^−4^ s^−1^ to 4.4 × 10^−4^ s^−1^ (Figure 7d). In contrast, the hydrogels with the branching crosslinkers PAsp-M20 and PAsp-M30 exhibited lower swelling rate constants than the minimum value of 2.4 × 10^−4^ s^−1^ observed for PEGDA575-crosslinked hydrogels (Figure 7e,f). The branching crosslinker not only enhanced the swelling ratio but also enabled the hydrogels to maintain a gradual absorption rate over time, offering a strategic approach to control the absorption behavior.

**Figure 8:**
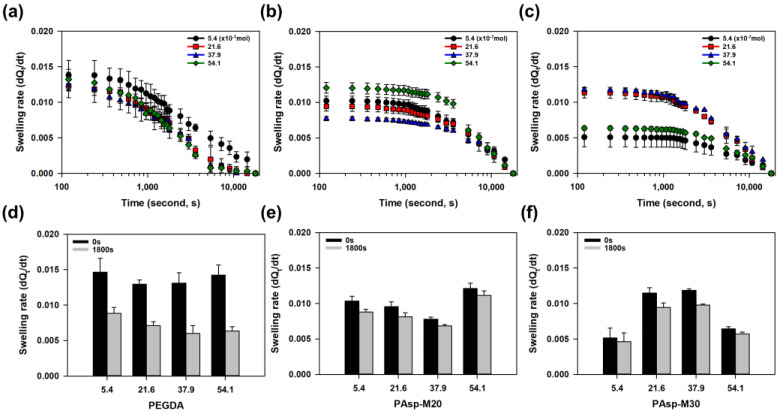
Real-time changes in swelling rate of the hydrogels were observed. (**a**) PEGDA575-crosslinked hydrogels exhibited a steep decrease in the initial swelling rate, which then led to a gradual reduction in the swelling rate over time (logarithmic scale). (**b**,**c**) Hydrogels incorporating branched crosslinkers showed a relatively slower initial swelling rate compared to PEGDA575-crosslinked hydrogels but demonstrated a gradual decline in swelling rate. (**d**) After 30 min, the swelling rate of PEGDA575-crosslinked hydrogels decreased by up to 55%, while (**e**,**f**) hydrogels with branched crosslinkers maintained swelling rates with a reduction of less than 18%.

The absorption behavior of hydrogels was analyzed through the swelling rate (Figure 8). As a result, the hydrogels crosslinked with PEGDA575 exhibited a rapid initial absorption rate, which sharply decreased and eventually led to a slow reduction in absorption rate (Figure 8a). In contrast, the hydrogels incorporating branched crosslinkers exhibited slower initial absorption compared to the PEGDA575-crosslinked hydrogels. However, the slower reduction in absorption rate allowed these hydrogels to demonstrate the potential for gradual and sustained absorption (Figure 8b,c). The absorption rate changes in each hydrogel were compared over the initial 0 to 1800 s. The PEGDA575-crosslinked hydrogels showed a rapid decrease in swelling rate of at least 40% to 55% within a short period (Figure 8d). However, hydrogels with branched crosslinkers demonstrated a slower rate of decrease, maintaining a more gradual decline in swelling rate, with a reduction of only about 18% compared to their initial swelling rate (Figure 8e,f). Thus, while the initial swelling rate of hydrogels incorporating branched crosslinkers was slightly lower, the swelling rate decreased gradually, allowing for the hydrogel’s progressive swelling behavior to be observed.

In this study, branched crosslinkers were synthesized by introducing acrylates with two different degrees of substitution (DS) into PSI precursors, and these crosslinkers were then incorporated into hydrogels to control their absorption behavior. To ensure a fair comparison, the same number of acrylates as in the control group, the conventional crosslinker PEGDA575, were used, thereby matching the number of crosslinking points in the final hydrogel. As a result, the hydrogels with branched crosslinkers demonstrated twice the swelling ratio and superior water absorption ability compared to the PEGDA575-crosslinked hydrogels. In addition, compared to the PEGDA575 group, which exhibited a rapid initial absorption followed by a sharp decrease, the hydrogels with branched crosslinkers showed relatively slower initial absorption but maintained a steady absorption rate with gradual decline, demonstrating continuous absorption behavior. Ultimately, the use of branched crosslinkers enhanced the swelling capability of the hydrogel and facilitated gradual swelling. This study successfully achieved both improved absorption capability and controlled absorption behavior by manipulating the structure of the crosslinkers. The branched crosslinker provides a structurally entangled state of polymer chains inside the hydrogel, and as these chains unravel during swelling, the hydrogel can continuously exhibit its potential absorption capacity [27]. The branched crosslinkers used in this study are expected to help avoid issues that can arise in the absorption behavior of particle aggregates composed of hydrogels with high swelling ratios. Moreover, it is anticipated that this new method will be effectively applied in existing industries to provide excellent swelling performance and continuous swelling behavior.

## 3. Conclusions

In this study, we analyzed the potential degradation of water absorption capacity in hydrogel particle aggregates with high swelling ratios using MRI and proposed a new strategy to address this issue by incorporating branched crosslinkers. The branched crosslinkers were synthesized by introducing acrylates with degrees of substitution (DS) of 20 and 30 into PSI precursors and then incorporated into hydrogels at the same acrylate concentration as the conventional crosslinker PEGDA575. As a result, the hydrogels with branched crosslinkers exhibited a two-fold increase in swelling ratio and a 50% decrease in swelling rate constant compared to PEGDA575-crosslinked hydrogels. Furthermore, while the PEGDA575-crosslinked hydrogels showed a rapid decrease in the swelling rate of up to 55% during initial absorption, the hydrogels with branched crosslinkers maintained a steady absorption rate with a gradual decrease of only about 18%, demonstrating continuous swelling behavior. Ultimately, we were able to create a hydrogel that not only exhibits excellent swelling ratio but also maintains a gradual swelling rate, enabling sustained absorption. This improvement is attributed to the entangled polymer network formed by the branched crosslinkers, which allows for a controlled and prolonged swelling process. This study introduces the branched crosslinker as an effective method for improving hydrogel absorption performance and controlling absorption rates, and it is expected to provide a new strategy for manufacturing high-quality hydrogels across various industries.

## 4. Materials and Methods

### 4.1. Materials

Aspartic acid, phosphoric acid, sulfolane, methanol, dimethylformamide, 2-amino ethyl methacrylate hydrochloride, ethyl ether, acrylic acid (AAc), poly(ethylene glycol) diacrylate 575 (PEGDA575), ammonium persulfate (APS), sodium hydroxide (NaOH), and N,N,N′,N′-tetramethylethylenediamine (TEMED) were purchased from Sigma-Aldrich (St. Louis, MO, USA).

### 4.2. Swelling Behavior of Hydrogel Particle Aggregates Analyzed by MRI

The swelling behavior of the hydrogel particle aggregates was analyzed using MRI (9.4T Animal Magnetic Resonance Imaging System, Varian, Palo Alto, CA, USA) to observe water movement. MRI, which utilizes proton resonance, has been employed to track water migration within hydrogels [24,25]. First, acrylic acid was dissolved in distilled water at a concentration of 32.5 wt%, and NaOH was added to neutralize 70 mol% of the acrylic acid. The amount of NaOH was added according to the molar ratio of acrylic acid. Subsequently, PEGDA575 was added as a crosslinker at concentrations of 0.1% and 1.0% by weight of the acrylic acid. A pre-gel solution was prepared by adding 2% APS (initiator) and 0.1% TEMED (catalyst). The pre-gel solution was placed between two glass plates with a 1 mm spacer and reacted for one hour to form a hydrogel of 1 mm thickness. The resulting hydrogel was cut into standard sizes, dried, and then pulverized using a mill. The hydrogel particles, with sizes around 100 μm, were obtained by sieving. To gradually observe the swelling behavior of hydrogel particle aggregates, an agarose well system was introduced as a release-controlled system instead of direct contact with water. Agarose wells are widely used in various biomedical applications and were prepared using the method from prior studies [28]. The agarose wells were dried to 90% of their weight before use. Dry hydrogel particles (22 mg) were placed in the agarose wells, which were then submerged in a 0.9% NaCl solution. The system was placed in an MRI machine, and images of the vertical and horizontal cross-sections were captured every 10 min. The MRI used the SEMS (spin-echo multi-slice) pulse sequence to convert proton density data into water distribution maps using specialized software. The SEMS pulse sequence was fixed with a repetition time ($T_{R}$) of 0.7 s and an echo time ($T_{E}$) of 10 ms. The images were acquired with a field of view of 3.0 × 3.0 cm and a resolution of 128 × 128 pixels. The resulting images were analyzed using Image J software (version 1.54m) to determine the intensity and density of pixels, from which the swelling behavior of hydrogel particle aggregates was analyzed.

### 4.3. Synthesis of Banched Poly(aspartice acid) Crosslinker, PAsp-g-MA

The branched crosslinker was synthesized by grafting acrylate onto an amino acid-based polymer backbone. First, 25 g of L-aspartic acid was dissolved in 125 g of sulfolane solvent, and 9.4 mmol of phosphoric acid was added as a catalyst. The reaction was conducted at 150 °C for 10 h. Afterward, the product was precipitated in excess methanol, washed with distilled water until the pH became neutral, and the precursor polymer, PSI (poly(succinimide)), was obtained [29,30]. The synthesized PSI was dissolved in dimethylformamide, and 2-aminoethyl methacrylate hydrochloride and triethylamine were added in a 1:3 molar ratio. The mixture was reacted under nitrogen for 24 h. The TEA-HCl salt formed during the reaction was removed by syringe filtration (0.45 μm, Nylon). The product was precipitated in excess ether, washed, and completely dried at room temperature to obtain PSI-g-MA. The degree of substitution (DS) of the synthesized PSI-g-MA was adjusted as shown in Table 1. The structure of the synthesized PSI-g-MA was analyzed by ^1^H-NMR (Avance II, Bruker, Billerica, MA, USA) at 400 MHz, using DMSO-*d_6_* as the solvent. The final product, PAsp-g-MA (poly(aspartic acid)-g-MA), was obtained by hydrating the PSI portion in a NaOH solution. The PAsp-g-MA was freeze-dried for further use. The branched crosslinkers synthesized with DS20 and DS30 were named PAsp-M20 and PAsp-M30, respectively. Table 1 includes a case where the acrylate substitution degree of the branched poly(aspartic acid) crosslinker was set to zero. As hydrogels are inherently defined by the presence of crosslinkers, PAsp (without acrylates) does not form a hydrogel network and was employed solely to observe the impact of crosslinking on hydrogel formation.

### 4.4. Preparation of Acrylic Acid Hydrogels with Branched Crosslinkers

The hydrogel was prepared using the branched crosslinker PAsp-g-MA and neutralized acrylic acid through free radical polymerization. Acrylic acid was dissolved in distilled water at a concentration of 32.5 wt%, and NaOH was added to neutralize 70 mol% of the acrylic acid. The amount of NaOH was added according to the molar ratio of acrylic acid. PEGDA575 was added at concentrations of 0.1%, 0.4%, 0.4%, and 1.0 wt% by weight of the acrylic acid. The amount of PEGDA575 added was calculated based on the weight of the acrylic acid. PAsp-g-MA was incorporated at the same acrylate molar concentration as PEGDA575, as calculated in Table 2. The pre-gel solution was prepared by adding 2.0% APS (initiator) and 0.1% TEMED (catalyst). The mixed pre-gel solution was placed between two quartz plates with a 1.0 mm spacer and polymerized for one hour. After polymerization, the quartz plates were removed, and the hydrogel was punched into 8.0 mm diameter discs using a biopsy punch (Kai Medical, Gifu, Japan).

### 4.5. In Situ Swelling Behavior Analysis of Hydrogels

The real-time swelling behavior of the synthesized hydrogels was evaluated by calculating the swelling ratio, a key indicator of the hydrogel’s water absorption capacity. The swelling ratio is defined as the weight ratio of the absorbed water to the dry weight of the hydrogel. To measure the real-time changes in the swelling ratio, the size of the hydrogel was monitored, and the expansion ratio was calculated. The expansion ratio is the ratio of the swelling degree of the hydrogel after complete swelling in the solvent to the initial size of the hydrogel immediately after fabrication [1,31].

(2) $$Expansion\;ratio={\left(\frac{Q_{f}}{Q_{i}}\right)}^{-\frac{1}{3}}-1=\frac{x_{f}}{x_{i}}-1$$

where $Q_{i}$ represents the degree of swelling of the hydrogel immediately after fabrication, and $Q_{f}$ represents the degree of swelling of the fully swollen hydrogel. This can also be calculated as the ratio of the length of the hydrogel immediately after fabrication ($x_{i}$) to the length of the fully swollen hydrogel ($x_{f}$). The degree of hydrogel swelling (Q) is defined as the volume fraction of the polymer in the hydrogel ($v_{2}$), calculated using the following equation [32,33]:

(3) $$Q={v_{2}}^{-1}=\rho_{P}\left[\frac{Q_{m}}{\rho_{S}}-\frac{1}{\rho_{P}}\right]$$

where $\rho_{s}$ is the density of water, and $\rho_{p}$ is the density of polymer in hydrogel. The swelling ratio ($Q_{m}$) of a hydrogel was determined by measuring the weight of fully swelled hydrogel and dried hydrogel and can be calculated using the following equation [34]:

(4) $$Q_{m}=\frac{W_{s}-W_{d}}{W_{d}}\times 100\left(\%\right)$$

where $W_{s}$ is weight of a fully swelled hydrogel, and $W_{d}$ is weight of dried hydrogel. In this study, to measure the hydrogel swelling at the same size, the hydrogel was immersed in saline (0.9 wt%) immediately after fabrication without the drying process, and its swelling was observed. Additionally, all hydrogels were used at the same size. To measure real-time swelling, the diameter of the swelling hydrogel was measured every hour to calculate the expansion ratio, from which the degree of swelling was determined. The diameter of the hydrogel was measured every 10 s for the first 3 min, every 1 min up to 10 min, every 10 min up to 60 min, every 30 min up to 3 h, and every 1 h up to 5 h after immersion. To analyze the swelling behavior of the hydrogel, the swelling rate ($r_{s}$) was calculated [12,35]. The swelling rate is derived from Fick’s law using the following relationship:

(5) $$r_{s}=\frac{dQ_{t}}{dt}=K_{s}\left(Q_{eq}-Q_{t}\right)$$

(6) $$\ln\left[\frac{Q_{eq}-Q_{0}}{Q_{eq}-Q_{t}}\right]=K_{s}t$$

where $Q_{eq}$ represents the degree of swelling of the fully swollen hydrogel, $Q_{t}$ denotes the degree of swelling of the hydrogel at a given time, $Q_{0}$ is the initial degree of swelling of the hydrogel, and $K_{s}$ is the swelling rate constant. In this study, the degree of swelling of the hydrogel immediately after fabrication was used as $Q_{0}$.

### 4.6. Statistical Analysis

Statistical significance was determined using one-way ANOVA followed by Tukey’s Multiple Comparison Test (* *p* < 0.1, ** *p* < 0.05, *** *p* < 0.01).

## Figures and Tables

**Table 1:** Characteristics of the synthesized branching crosslinkers.

Sample	Feed ^a^	DS ^b^	Number ^c^
PSI	100/0	-	-
PAsp-M20	80/20	13	25
PAsp-M30	70/30	17	33

^a^ Feed mol ratio (mol%, succinimide unit/methacrylate). ^b^ Degree of substitution (mol%) determined based on ^1^H-NMR of graft copolymer. ^c^ Number of methacrylate per one polymer chain.

**Table 2:** Amount of PAsp-g-MAs introduced into hydrogel compared to PEGDA.

PEGDA575 (*w*/*w*%)	Number of Acrylates (×10^−7^ mol%)	PAsp-M20 (*w*/*w*%)	PAsp-M30 (*w*/*w*%)
0.1	5.41	0.3	0.26
0.4	21.6	1.3	1.0
0.7	37.9	2.2	1.8
1.0	54.1	3.1	2.6

## Data Availability

Data are contained within the article.
